# Current Strategies for Engineered Vascular Grafts and Vascularized Tissue Engineering

**DOI:** 10.3390/polym15092015

**Published:** 2023-04-24

**Authors:** Jun Chen, Di Zhang, Lin-Ping Wu, Ming Zhao

**Affiliations:** 1 Department of Organ Transplantation, Zhujiang Hospital, Southern Medical University, Guangzhou 510280, China; 2 Center for Chemical Biology and Drug Discovery, Laboratory of Computational Biomedicine, Guangzhou Institute of Biomedicine and Health, Chinese Academy of Sciences, Guangzhou 510530, China

**Keywords:** engineered vascular grafts, vasculature, vascularized tissue engineering, vascularization

## Abstract

Blood vessels not only transport oxygen and nutrients to each organ, but also play an important role in the regulation of tissue regeneration. Impaired or occluded vessels can result in ischemia, tissue necrosis, or even life-threatening events. Bioengineered vascular grafts have become a promising alternative treatment for damaged or occlusive vessels. Large-scale tubular grafts, which can match arteries, arterioles, and venules, as well as meso- and microscale vasculature to alleviate ischemia or prevascularized engineered tissues, have been developed. In this review, materials and techniques for engineering tubular scaffolds and vasculature at all levels are discussed. Examples of vascularized tissue engineering in bone, peripheral nerves, and the heart are also provided. Finally, the current challenges are discussed and the perspectives on future developments in biofunctional engineered vessels are delineated.

## 1. Introduction

Biological processes, such as tissue regeneration, stem cell maintenance, and cancer progression, can be regulated by vasculature and its surrounding microenvironment through distinct biophysical and biochemical cues, as well as the interference of cell behavior and cell–cell communications [1,2,3,4,5]. Vasculature exhibit tissue-specific phenotypes, varying from molecular signatures to architectures and functionalities in different organs [6]. Vessel networks not only play an important role in the transportation of oxygen and nutrients, but also regulate the deposition and remodeling of the peripheral ECM through signaling between vascular and nonvascular cells [7,8].

In general, three distinct processes are responsible for vascularization: vasculogenesis, angiogenesis, and arteriogenesis [9,10]. Vasculogenesis is a process including neovessel formation associated with embryogenesis, while angiogenesis describes the formation of vasculature from existing and mature vessels; finally, arteriogenesis indicates the consolidation and remodeling of existing collateral vessels [9]. Cell signature, biochemical, and biophysical cues that regulate vascularization in these three mechanisms have been studied to guide the in vitro formation of vascular grafts of various diameters, as well as interconnected vasculature. However, the complexity of the vascularization process and technical challenges currently limit most of these efforts in the preclinical stage.

**Figure 1:**
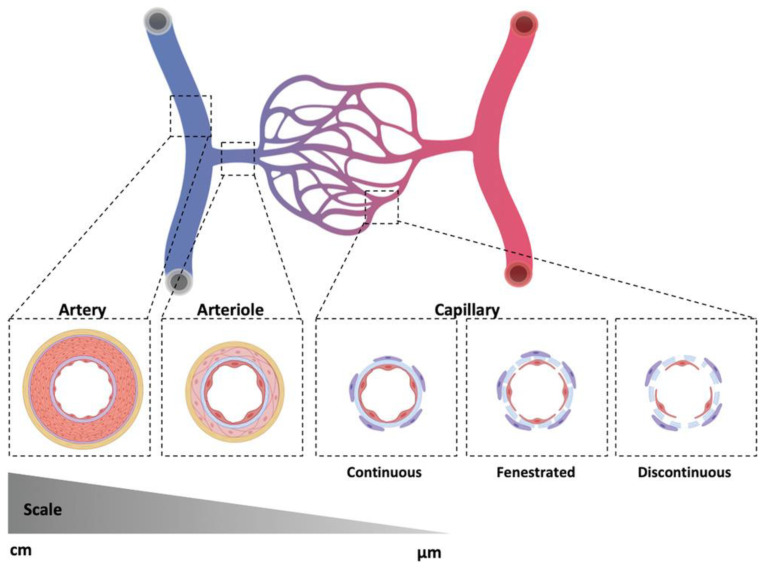
Architecture of artery, vein, and capillary [6]. Copyright 2020, Wiley-VCH.

Blood vessels can be categorized into three types: arteries, veins, and capillaries (Figure 1). Each type of blood vessel has its own unique functionality and structure. Arteries and veins are responsible for the transportation of oxygenated or deoxygenated blood from or to the heart, respectively. They are composed of a tunica internal, which supports the vascular endothelium surrounded by one or more layered smooth muscle cells (SMCs), tunica media, and collagen fibril tunica externa. Arteries usually possess thicker tunica media compared to veins to withstand high pressure. Capillaries connect arteries and veins and are responsible for the exchange of oxygen, nutrients, and waste in each organ. The walls of capillaries are only one cell in thickness, and are surrounded by a thin vasal lamina and scattered pericytes to facilitate solute transportation. For different purposes, multiple types of engineered vascular grafts have been developed. Tubular grafts of varied diameters were fabricated as substitutes for blocked arteries or veins, while capillary networks and hierarchical vasculature were developed for prevascularized tissue engineering or the regeneration of ischemic tissues. A comprehensive understanding of vascular biology in different tissues facilitates the development of vascularized tissue engineering. Recapitulating the architecture of native vascular networks may pave a new path for engineering large-scale tissues and even organs.

In this review, we briefly introduce the categories of engineered vascular grafts, and then summarize the materials and techniques used for their fabrication. Then, we discuss the relationship between neovascularization and tissue regeneration in peripheral nerves, bone, and the heart, and summarize the current vascularization strategies in these engineered tissues. Finally, we discuss the current challenges and delineate our perspective on the future development of biofunctional engineered vessels.

## 2. Tissue-Engineered Vascular Grafts

Cardiovascular diseases, including, but not limited to, coronary heart disease (CHD) and peripheral arterial disease (PAD), are the principle cause of death worldwide, especially in developed countries, where cardiovascular diseases affect more people than all types of cancer combined [11,12]. Common disorders such as myocardial infarction (MI) and critical limb ischemia happen as a result of the occlusion or stenosis of blood vessels; thus, the prompt restoration of local blood perfusion is required to prevent heart failure or to aid in the repair of ischemia [13,14,15]. A large diameter (>8 mm) and medium to large diameters (6–8 mm) are usually needed for aortic, iliac, and femoral artery repairs, while small-diameter (1–6 mm) vascular grafting is required for coronary artery replacement caused by atherosclerosis or embolism [6].

Most tissues in the body are nourished by hierarchical tree-like vascular structures with complex and diverse branching configurations. Cells embedded in the biological environment cannot obtain vital nutrients and oxygen, or dispose of metabolic waste without a proper vascular network; e.g., occlusion in mesovasculature (50 μm–1 mm) or microvasculature (<50 μm) can lead to cell death and subsequent tissue necrosis, or even complete tissue dysfunction [16,17]. For tissue engineering thicker than the diffusion limitation distance (100–200 μm), a prevascularized network is required to facilitate the transportation of nutrients and waste in cell-dense constructs [18,19,20]. Hierarchically connected arteriole-sized and capillary-sized built-in vasculature can mimic natural vasculature in tissues, and anastomosis between the engineered constructs and the host is required for the rapid functionalization of engineered tissues after implantation [21,22,23,24].

### 2.1. Middle- and Large-Diameter Artificial Grafts

Large-diameter artificial grafts were firstly developed due to their fabrication being less complex [25]. Synthetic nonbiodegradable polyethylene terephthalate (PET, Dacron^®^) was used for the first artificial vascular graft, and was successfully implanted in a human in 1954 by Barkey [26,27]. Since then, a variety of materials, including PET, expanded polytetrafluoroethylene (ePTFE), and polyurethane (PU), have been clinically approved and/or used in clinical trials for commercial vascular grafts as substitutes to autologous vascular grafts [28,29,30]. Due to the high blood flow rates in large vessels, the risk for occlusion is relatively low, and satisfactory results with a patency of 85–90% at 5 years and 75–80% at 10 years were obtained after long-term implantation [25,31]. Medium- to large-diameter vascular grafts have demonstrated more than a 50% patency within 15 years of implantation [30,32,33,34]. However, commercial synthetic grafts are much stiffer and highly hydrophobic compared to native vessels, leading to poor interactions with vascular cells and subsequent reduced biocompatibility [35,36,37]. In addition, polymeric materials are susceptible to bacterial infection, which can lead to graft rejection due to an inflammatory response being evoked [38].

Accessorial modifications to luminal surfaces have been developed to improve biocompatibility and clinical success rates. Bioactive molecules, including heparin [39,40], gelatin [41,42], dopamine [41,43,44], collagen [45,46,47], RGD peptides [48], etc., were immobilized to enhance cell adhesion, proliferation, and reendothelialization by mimicking the natural extracellular matrix (ECM), achieving improved patency rates, reduced bleeding, and the prevention of graft collapse [49,50].

### 2.2. Small-Diameter Artificial Grafts

Even though synthetic vascular grafts have demonstrated satisfactory outcomes in large- and middle-diameter vascular repairs, polymeric small-diameter grafting can only maintain 39% patency at 5 years, since they fail to recapitulate the key elements of the native vasculature [51,52]. The rough and hydrophobic luminal surface of commercial synthetic grafts fails to allow endothelial cells (ECs) to attach, proliferate, and form a confluent endothelium, which can resist thrombus formation by preventing platelets and red blood cells from adhering to endothelial cells via a barrier composed of glycocalyx [53,54]. Instead, increased interactions with blood cells and protein adsorption take place, accelerating clot formation, and SMCs migration to the intima, inducing intimal hyperplasia, which is the major cause of mid-to-late graft failure [55,56].

Although plenty of studies aiming at capturing the structural and functional properties of native arteries have been carried out, there have been no small-diameter synthetic grafts approved yet for clinical use at this time. In addition, current small-diameter revascularization exclusively relies on autologous grafts [57,58]. Tissue-engineered vascular grafts need to replicate the major features of native blood vessels for long-term functionality and avoid possible complications, including thrombosis, graft failure, and aneurysms [59]. First of all, the mechanical properties of grafts need to combine high strength with elasticity to withstand high-pressure flow without rapture or deformation, and to retain integrity after anastomotic sutures [60]. Secondly, essential biocompatibility is required for EC attachment, proliferation, and endothelium formation to resist thrombosis and intimal hyperplasia [54,61,62]. Thirdly, implanted grafts should maintain a long-term matching to surrounding blood vessels [63]. For example, for nondegradable vascular grafts, the mechanical properties should be kept as the value immediately after fabrication to avoid dilation, delamination, and graft rupture, while the compensation of newly formed ECMs should match the biomaterial digestion for biodegradable tissue-engineered grafts with no aneurysms or intimal hyperplasia development during tissue remodeling [64]. Finally, the production and sterilization process must be facilely expanded from laboratory to industry scale, providing a versatile off-the-shelf small-diameter vascular graft product [56].

### 2.3. Engineered Vasculature

A dense network of capillaries within 100 μm from one another provides optimal conditions for the transportation of gasses, nutrients, metabolites, and circulating cells to tissues in the body [6,65]. Previous works have demonstrated that prematured microvasculature can improve overall in vivo tissue regeneration, organization, and functionality through the construct–host microvascular integration upon implantation [21,66,67,68]. Various strategies for appropriate revascularized networks in thick engineered constructs have been explored, including optimizing the micro–nanostructure, morphology, porosity, and roughness of the extracellular environment, and introducing biochemical and/or mechanical stimuli [69,70,71,72,73]. However, engineered microvasculature formed through self-assembling are hard to surgically integrate to a host’s vascular network [74]. The ideal vasculature for thick engineered tissue should contain seamlessly connected hierarchical meso- and microvasculature, of which the diameter should follow Murray’s law [75]. The perfusion of vasculature should be achieved as soon as possible, either through surgically or spontaneously anastomosing the mesoscale vessels with the host’s blood vessels [21,22,76].

## 3. Material Selection

Both synthetic and natural polymers have been used for the production of vascular scaffolds. Synthetic polymers, including polyesters, polyethers, and poloxamers, have been widely used for vascular scaffolds due to their adjustable mechanical strength and degradation rates, thermal stability, and well-established fabrication techniques [77]. However, the lack of cell binding sites and high hydrophobicity of synthetic polymers can impair endothelial cell adhesion and proliferation, as well as causing platelet aggregation and intimal hyperplasia [21,78,79,80].

Natural polymers, such as proteins and polysaccharides, have also been utilized for the development of engineered vessels. Natural polymers generally exhibit more similar properties to native ECMs by providing cell recognition and adhesion domains. Despite having bioactive properties superior to synthetic materials, unmodified natural polymers crosslink mostly via physical interactions, and exhibit poor mechanical properties and rapid degradation [81]. Moreover, a limited supply, batch-to-batch variations, and potential cross-contamination hinder them from reaching off-the-shelf use [82,83]. In order to improve the properties of fabricated vasculature, advances in the isolation, purification, and manufacturing processes of natural polymers are required, and modifications with synthetic functional groups could enhance the mechanical strength to match the mechanical properties of native vessels [56,84].

### 3.1. Synthetic Polymers

Synthetic materials used for engineered vascular scaffolds can be classified into two categories: nondegradable and biodegradable polymers. Large-, medium-, and small-diameter tissue-engineered vascular grafts composed of each category have been reported since the 1950s [85].

#### 3.1.1. Nondegradable Polymers

Nondegradable polymers, such as ePTFE and PET, have mostly been used in the creation of medium- and large-diameter artificial blood vessel grafts, which have been widely applied in human treatments since the 1970s [86]. The electronegativity and biostability of ePTFE and PET can minimize thrombosis and restenosis in the case of high rates of blood flow and large flow areas in large- and medium-diameter vessels. However, a few of them have been used as vascular scaffolds of <6 mm. Unsatisfactory results, such as cell hyperplasia and the eventual failure of these vascular grafts, were obtained because of their high hydrophobicity, mismatches between the materials and native vessels, and the induced local chronic foreign body response, resulting in thrombi formation [87,88,89]. Alterations have been determined to increase the hydrophilicity to in turn enhance cell adhesion and proliferation [90]. Chen et al. embedded ECM and CD34 in ePTFE to facilitate endothelization and decrease platelet adhesion [91].

#### 3.1.2. Biodegradable Polymers

Compared to nondegradable polymers, biodegradable polymers exhibit better biocompatibility with suitable degradation rates.

##### PGA

**Figure 2:**
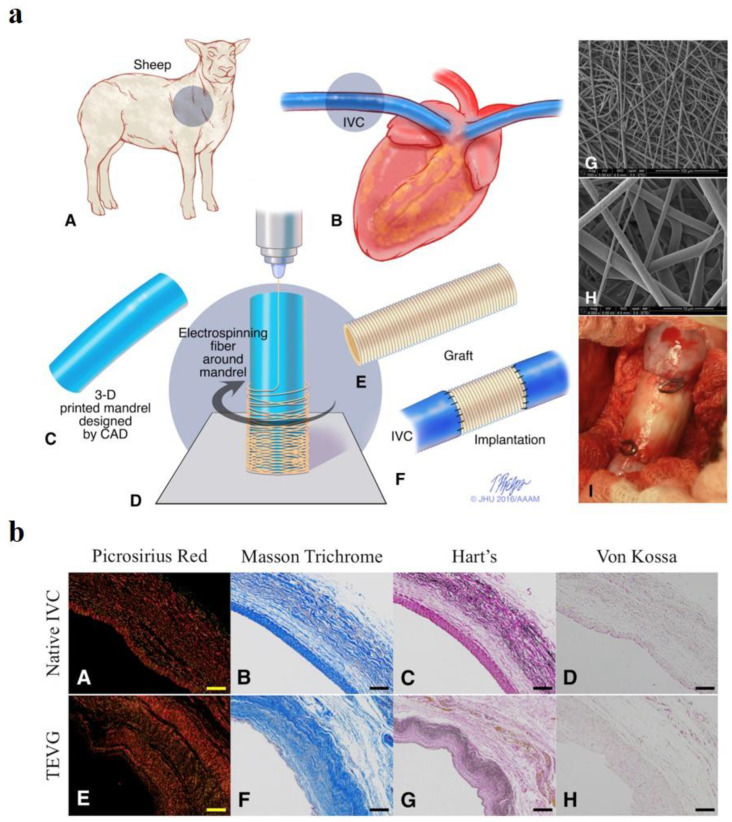
Preclinical study of cell-free 3D-printed nanofiber vascular grafts. (**a**) Study design of cell-free patient-specific nanofiber tissue-engineered vascular graft. (**A**,**B**) Acquiring the topographical information of the blood vessel from a preoperative angiogram in a sheep model. (**C**) Three-dimensional-printed mandrel designed with computer-aided design (CAD). (**D**) Electrospinning fibers around mandrel to fabricate nanofiber vascular grafts. (**E**) Nanofiber vascular graft. (**F**) Implantation of fabricated grafts. (**G**,**H**) SEM images of nanofibers. (**I**) Image of the implanted vascular graft. (**b**) Collagen and elastin deposition at 6 months postimplantation. *Picrosirius red* (**A**), Masson’s trichrome (**B**), Hart’s (**C**), and von Kossa (**D**) stainings of native inferior vena cava (native IVC) at 6 months. *Picrosirius red* (**E**), Masson’s trichrome (**F**), Hart’s (**G**), and von Kossa (**H**) stainings of tissue-engineered vascular graft (TEVG) at 6 months. Ref. [96] Copyright 2017, Elsevier.

Polyglycolic acid (PGA) is a highly flexible polyester, with a degradation rate of approximately 6–8 weeks, inducing little inflammatory response [92,93]. It has been reported that PGA vascular grafts were able to remain patent up to 4 weeks after implantation in vivo [94]. The in vivo degradation rate is too fast to match the regeneration rate for clinical applications. Other polymers, such as polylactic acid (PLA), poly (lactide-co-caprolactone) (PLCL), and poly (DL-caprolactone-co-lactic acid) (PCLLA), were added to slow the in vivo degradation rate, introducing satisfactory remodeling observed up to 6 months (Figure 2) [95,96,97]. Niklason et al. seeded vascular smooth muscle cells (VSMCs) onto PGA conduits and cultured them with a pulsatile flow for 8 weeks in vitro. The full maturation of the vascular grafts was achieved by replacing the PGA scaffolds with VSMC-secreted collagen matrix deposition [94,98,99]. After decellularization, the engineered blood vessels were tested in multiple small and large animal models, showing over an 88% patency in 3.5 mm sized grafts up to 6 months [100,101]. The engineered human acellular vessels were named as Humacyte (Humacyte Inc., NC, USA), with clinical trials being underway since 2012 [74,102,103].

##### PLA

PLA is a nonimmunogenic polyester approved by the FDA. The degradation rate of PLA is relatively slow (6–8 months). However, the highly hydrophobic surface of PLA impairs cell development, and results in the unsatisfactory performance of vascular scaffolds composed of only PLA [104]. A copolymer of PLA and polycaprolactone (PCL) was developed to adjust the biodegradation rates, biocompatibility, and elasticity for facilitating endothelial cell adhesion and proliferation in vitro, as well as endothelization and collagen fiber deposition in vivo [105,106]. Combining PLA with bioactive molecules such as heparin, collagen, and chitosan through electrospinning can greatly increase the infiltration and attachment of endothelial cells, thus, improving the hemocompatibility of the vascular grafts [104,107]. Hashi et al. reported that by seeding bone-marrow-derived stem cells (BMDSCs) onto PLA vascular scaffolds, the patency rates could remain at nearly 100% after 60 days postoperative in a rat model [108].

##### PCL

**Figure 3:**
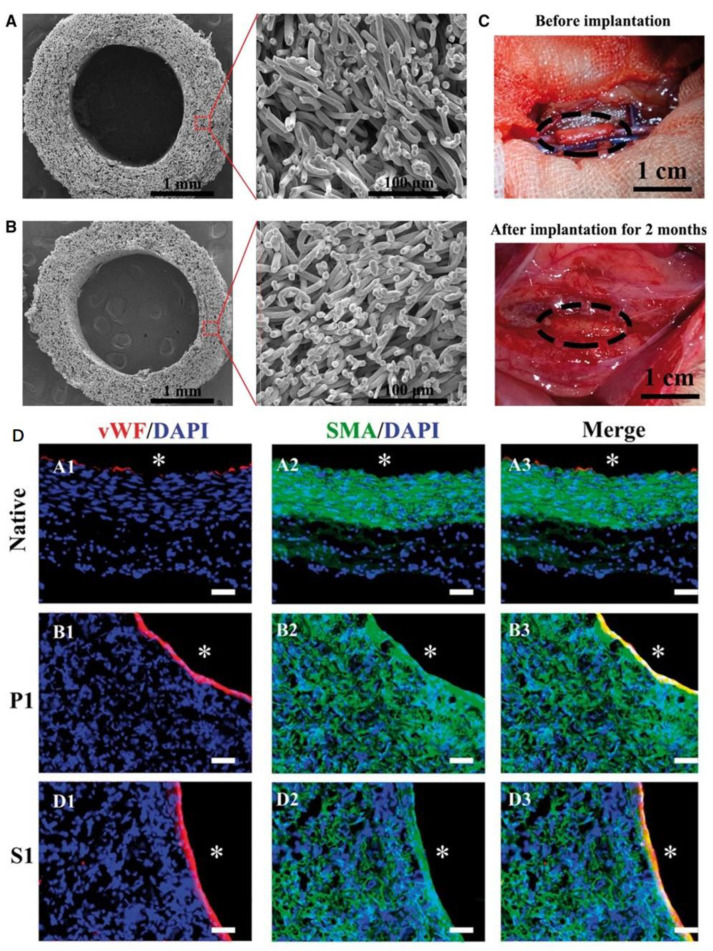
In vitro and in vivo characterization of the PCL and heparin-modified PCL vascular grafts. (**A**,**B**) SEM images of PCL and heparin-modified PCL vascular grafts. (**C**) Optical images of scaffold before and after implantation. (**D**) Endothelialization (vWF) and smooth muscle regeneration (SMA) in the grafts after 1 month. (**A1**–**A3**) native vascular, (**B1**–**B3**) PCL implanted after 1 month, (**D1**–**D3**) heparin-modified PCL implanted after 1 month. Graft lumen was indicated by *. Ref. [111] Copyright 2018, Oxford University Press.

PCL serves as the most commonly used polymer in vascular scaffolds due to its excellent biocompatibility, suitable in vivo degradation rates, low cost, mature fabrication techniques, and off-the-shelf properties [109]. Nevertheless, the performance of PCL grafts is similar to ePTFE grafts in patency rates, in spite of their superior biocompatibility, faster endothelization, and less-consequential stenosis-free rate [110]. By increasing the porosity or reducing the hydrophobicity by blending natural polymers, the mechanical strength of PCL scaffolds can be adjusted to those of the native artery (1–2 MPa), and reendothelialization can be promoted without blood coagulation (Figure 3) [111,112,113].

##### PGS

Poly (glycerol-co-sebacate) (PGS) is a highly biocompatible but rapidly bioabsorbable elastomer. The degradation rate of PGS is similar to PGA, while the hemocompatibility of PGS is superior, as it can not only facilitate the proliferation of ECs and the infiltration of VSMCs, but also prevent platelet adhesion and inflammation [114,115,116,117]. Khosravi et al. used PGS as the inner layer with a PCL outer sheath, which exhibited excellent long-term patency, a mild inflammatory response, and the regeneration of organized ECs and SMC constructs [118].

##### PEG

**Figure 4:**
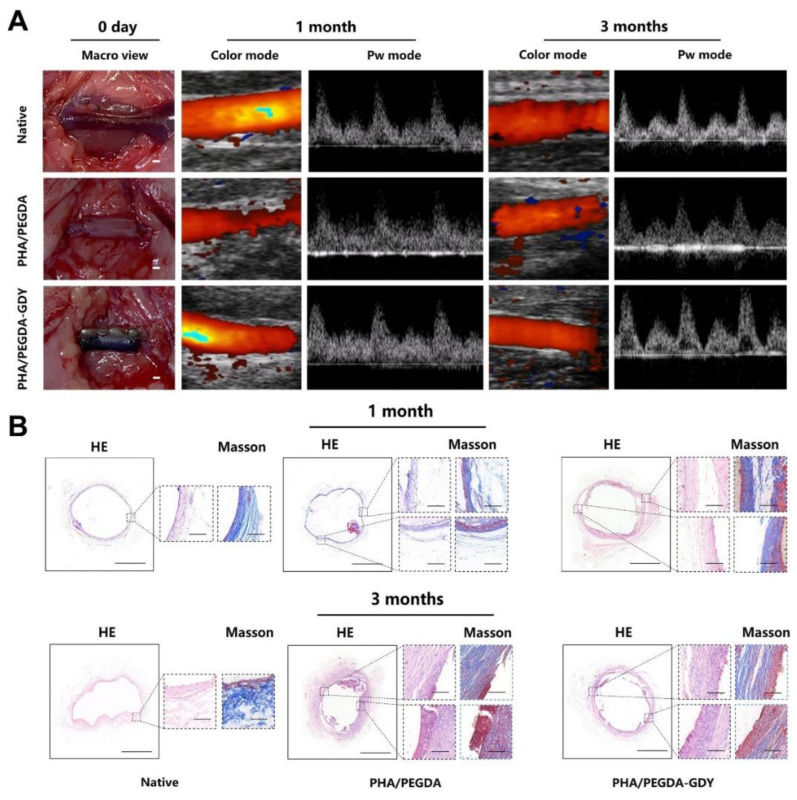
In vivo implantation of PEGDA-based vascular grafts. (**A**) Microscopic views after implantation and ultrasound images of native blood vessels (native), PHA/PEGDA grafts (PHA/PEGDA), and PHA/PEGDA grafts with uniformly dispersed graphdiyne (PHA/PEGDA-GDY). (**B**) HE and Masson staining results of implanted grafts. Ref. [123] Copyright 2022.

Polyethylene glycol (PEG) is a nontoxic biodegradable polymer approved by the FDA due to having nonimmunogenic and nonantigenic performance in biological applications [119]. Diacrylate-derived PEG (PEGDA) maintains the nonthrombogenic and hyperplasia reducing properties of PEG and is able to gel rapidly in the presence of photoinitiators and light irradiation [120,121]. Hahn et al. developed a tissue-engineered vascular scaffold by enveloping smooth muscle progenitor cells within a PEGDA hydrogel decorated using adhesive ligands and collagenase degradable sequences [122]. After culturing in vitro for 8 weeks, the elastin and collagen deposition reached the native arterial favorable range [122]. Hou et al. developed amphiphilic and fatigue-resistant small-diameter vascular grafts by combining PEGDA with polyhydroxyalkanoates (PHAs) [123]. High patency rates were achieved 3 months postoperation, and cell infiltration, as well as remarkable tissue regeneration, was observed (Figure 4) [123].

### 3.2. Natural Polymers

Natural components of the ECM have attracted increasing attention as alternatives for less bioactive synthetic grafts. Proteins and polysaccharides, such as collagen, gelatin, elastin, fibrin, chitosan, alginate, and cellulose, possess better biocompatibility and bioactivity due to the remaining biomimetic binding sites for cell development that promote the recruitment, colonization, and proliferation of intrinsic cells [124,125,126,127,128].

#### 3.2.1. Collagen and Gelatin

**Figure 5:**
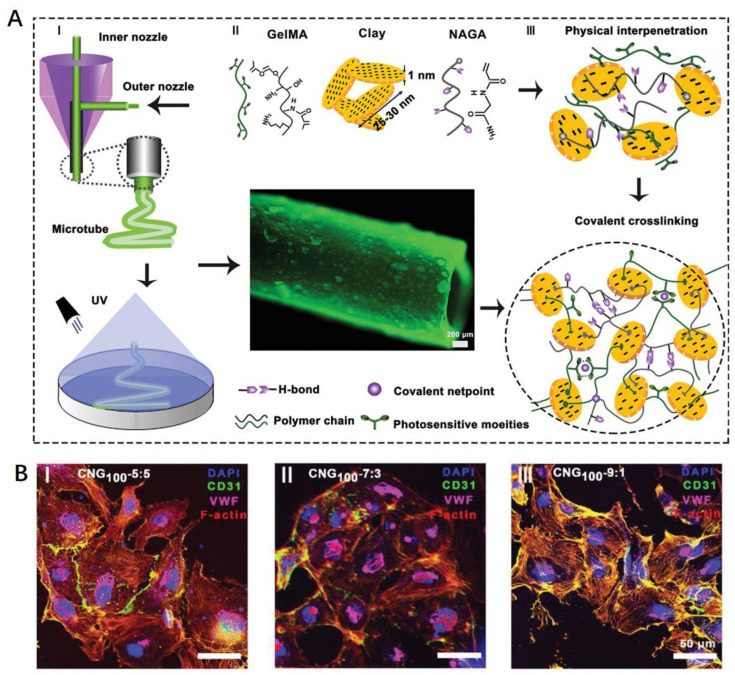
GelMA-based vascular microtubes. (**A**) Coaxial extrusion printing of microtubes. (**B**) Biocompatibility and endothelialization of hydrogel microtubes. Ref. [137] Copyright 2020, Wiley-VCH.

Collagen and its derivative gelatin are the most used natural polymers in vascular tissue engineering. Collagen is the main component of the ECM in most tissues, including blood vessels [129]. However, the in vivo degradation rate of collagen is approximately 5 h, which is too short for clinical applications [130]. Crosslinked collagen conduits after dehydration can match a mammalian vein value with a burst pressure of ~1300 mmHg and a compliance of 1.7%/100 mmHg, but a low strength at anastomosis interfaces [131]. Circular electronspun meshes of collagen were developed by Zhang et al., possessing a similar dynamic compliance and increased rupture force to saphenous veins [132]. Collagen and gelatin were also used in combination with synthetic polymers for vascular scaffold fabrication through electrospinning [42,133]. By altering the concentration of natural proteins, the hemocompatibility and mechanical strength can be balanced for vascular cell growth and a reduction in the local inflammatory response [109]. In order to increase the mechanical strength of the grafts, gelatin was modified with methacrylate groups to form a semisynthetic gelatin methacryloyl (GelMA), which could maintain the bioactivity of gelatin with adjustable mechanical properties [134,135]. The cell-attached peptide motifs of GelMA hydrogels mimic the essential properties of native ECMs for cell proliferation and spread, while the metalloproteinase-responsive peptide motifs lead to dilation and a decrease in mechanical strength after in vivo implantation [136]. Liang et al. developed a series of diameter-tunable microtubes with enhanced mechanical strength by blending a H-bonding monomer N-acryloyl glycinamide nanoclay with GelMA (Figure 5) [137]. The coaxial printed vascular grafts exhibited a Young’s modulus of ~21 MPa, a burst pressure of ~2500 mmHg, a suture retention strength of ~280 gf, and an antifatigue performance of ~200 cycles [137].

#### 3.2.2. Elastin

**Figure 6:**
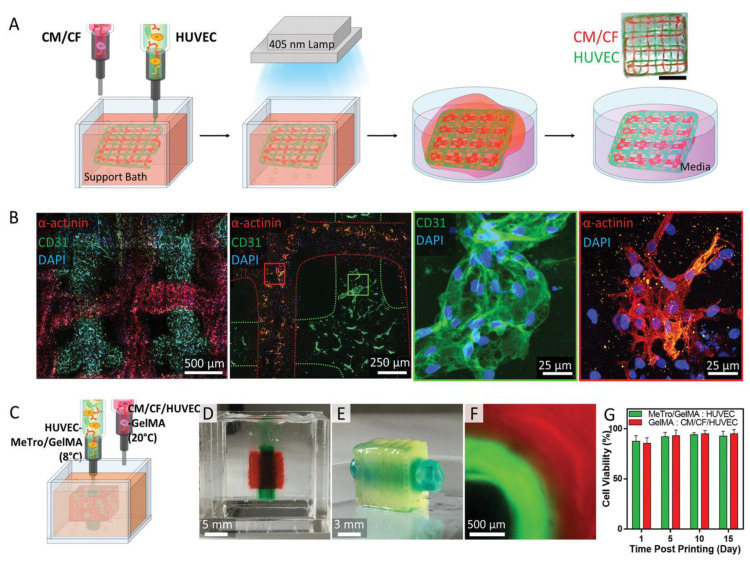
Cell-laden elastic constructs using modified human-recombinant elastin. (**A**) A schematic illustration of cell-laden 3D printing. (**B**) Immunostaining of the printed structure at 7 days after printing. (**C**) Scheme of vascularized tissue printing. (**D**–**F**) Optical and fluorescence images of printed vascularized tissue. (**G**) Cell viability after printing. Ref. [142] Copyright 2020, Wiley-VCH.

Elastin is another predominant component of the ECM in arterial tissues. It is able to promote SMCs’ contractility and introduce aligned collagen fiber deposition that mimics the construction of the native ECM [138,139]. Elastin was used in association with PCL by Wise et al. to enhance its patency and mechanical properties. However, the durability was just like the human internal mammary artery tested in a rabbit model [140]. Elastin has been proved to play a significant role in aortic morphogenesis and the prevention of intimal hyperplasia in native tissue, while only a little in vivo research has been carried out due to its limited resources, high expense, and batch-to-batch variations [141]. Lee et al. modified human-recombinant elastin with methacrylic groups and fabricated it into vascular scaffolds via extrusion printing with GelMA (Figure 6) [142]. The endothelium barrier was observed in the printed vascular grafts to have a minimal inflammatory response and efficient biodegradation in vivo.

#### 3.2.3. Silk Fibroin

Silk fibroin (SF) proteins are natural fibers characterized by mechanical hardness, processing malleability, self-assembly, and biocompatibility [143]. Different from collagen and elastin, SF can be produced into nanofiber tubes alone or in combination with bioactive macromolecules as support [144,145,146,147]. Marelli et al. developed a SF tubular scaffold coated with collagen that exhibited a physiologically relevant burst pressure (894 ± 24.91 mmHg) and compliance (3.24 ± 0.58%/80–120 mmHg) [147]. Liu et al. blended heparin into the inner layer of SF tubes; the bilayered grafts showed successful SMCs and fibroblast infiltration in the outer layer as well as low thrombogenicity on the luminal surface [148]. Cattaneo et al. engineered SF scaffolds through electrospinning and in vivo tests, showing that elastic lamina and vasa vasorum could be formed within 7 days in a rat abdominal aorta [149]. Filipe et al. found near-complete endothelialization by 6 weeks postsurgery in a rat aortic interposition grafting model [35], and Enomoto et al. reported that small-diameter SF-based grafts (1.5 mm diameter) retained an 85% patency at 1 year after implantation [150].

#### 3.2.4. Chitosan

Chitosan is a mucoadhesive and biodegradable polysaccharide that is the second most used natural polymer next to collagen in vascular tissue engineering [58,151]. Chitosan has a similar structure to glycosaminoglycans (GAGs) in the ECM and exhibits biodegradability, anticoagulation, anti-inflammatory, and antimicrobial properties [152]. Chitosan has been used for the surface modification of several polymer-based vascular scaffolds, with its cationic property enhancing cell adhesion, morphology, and growth [153,154,155]. Chitosan-based tubular scaffolds have also been fabricated, with their porosity structure providing a favorable microenvironment for cell infiltration and tissue integration [156,157]. Aussel et al. engineered a vascular scaffold with chitosan through sequential NaOH-induced gelation and gaseous NH_3_-induced gelation [158]. By modulating the chitosan concentration, the mechanical properties of the tubes could reach a suture retention strength of 1.1 ± 0.5 N, a compliance of 11.50 ± 3.90%/100 mmHg, and a burst pressure of 1313 ± 196 mmHg [158]. Yan et al. fabricated a vessel-like-structured chitosan hydrogel via a templated electrodeposition, of which the diameter could be lowered to 400 μm in an ambient environment [159].

#### 3.2.5. Alginate

**Figure 7:**
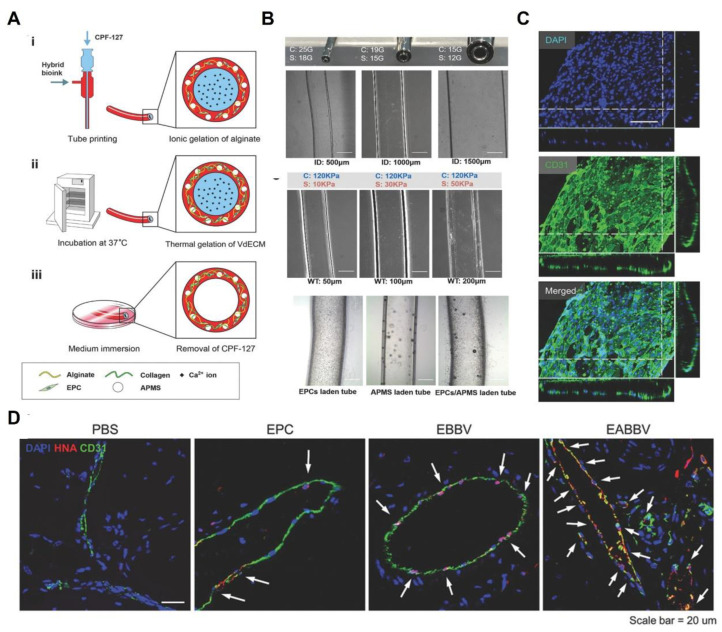
Alginate-based vascular grafts. (**A**) Scheme of coaxial printing of EPCs and atorvastatin-laden blood vessels. (**B**) Optical images of coaxial needles and microscopic images of alginate tube, EPC-laden tube, and EPCs/atorvastatin-laden tube. (**C**) Endothelialization of the engineered vessels. (**D**) Immunostaining images of implanted tubes. Ref. [163] Copyright 2017, Wiley-VCH.

Alginate is an anionic polysaccharide composed of guluronic acid and mannuronic acid [160]. Alginate can go through ionotropic gelation in the presence of divalent ions such as Ca^2+^. However, there are no cell-adhesive ligands on it before chemical modification. Cell-adhesive motifs, such as RGD peptides, gelatin, heparin, etc., were used to modify alginate to optimize the microenvironment for cell development [161]. Gao et al. incorporated decellularized ECMs into an alginate hydrogel and developed a series of vascular tubes through the coaxial printing technique [162,163,164]. It was reported that the engineered EC-laden grafts exhibited not only an endothelium maturation, but also selective permeability and antithrombogenicity [164]. Moreover, when combined with an atorvastatin delivery microvehicle, endothelial progenitor cells (EPCs) could quickly eliminate local ischemia in a mouse model (Figure 7) [163].

## 4. Fabrication Methods for Tissue-Engineered Vascular Grafts

### 4.1. Biobased Techniques

#### 4.1.1. Decellularization

**Figure 8:**
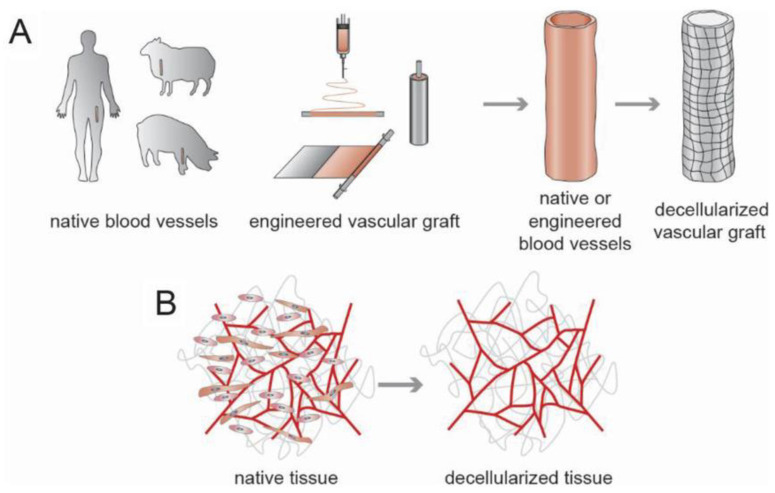
Strategies for fabrication of decellularized vascular grafts [64]. (**A**) Scheme for decellularized vascular grafts in large scale. (**B**) Scheme for decellularized vasculature grafts. Copyright 2019, Wiley-VCH.

To obtain decellularized scaffolds that can maintain the native architecture and composition of blood vessels, cellular components were removed through the use of detergents, chelators, enzymes, freeze/thawing, agitation, or their combinations [6,165,166,167]. A decellularized ECM (DECM) possesses biochemical and mechanical cues that can promote cell adhesion, proliferation, differentiation, and tissue organization (Figure 8) [64,168]. Allogenic human DECM vessels have demonstrated successful clinical outcomes in clinical trials [169]. However, the lack of sources and the potential pathogenicity prevent it from off-the-shelf applications. The xenogenic DECM has a prolific source and exhibits similar or increased compliance and burst pressure compared to human-native vessels [25,170,171], while the incomplete removal of cellular components and immunological ECM components can elicit a severe inflammatory response, which can lead to acute thrombosis and calcification [169,172]. Surface modifications were used to improve the endothelialization of the DECM scaffolds. Dimitrievska et al. deposited heparin as a continuous shielding layer to the underlying thrombogenic ECM, reducing platelet adhesion and improving blood compatibility [173]. Jiang et al. decorated DECM grafts with an antioxidant poly (1, 8-octamethylene-citrate-co-cysteine) (POCC), achieving reduced oxidative tissue damage and calcification [174]. Pre-endothelium through recellularization with ECs, bone-marrow-derived cells, and genetic knockout cells can greatly reduce thrombosis formation and increase the long-term patency up to 60% [175,176,177].

Decellularized engineered scaffolds have also been fabricated. There are two strategies mostly used to produce cell-laden engineered grafts before decellularization: one is culturing cells on native or synthetic polymer scaffolds to produce an adequate ECM [100,178], and the other is to embed polymeric rods in vivo to form an autologous fibrocellular tissue capsule [88,179]. Syedain et al. cultured fibroblasts in a fibrin tube and disposed of the cellular components to acquire the ECM-deposited grafts [178]. The implanted scaffolds showed fibroblast and SMC infiltration in vivo with collagen and elastin deposition. In addition, the diameter and volume increased with the growth of the host without rupture, demonstrating successful in vivo remodeling. Geelhoed et al. subcutaneously implanted copolymer poly(ethylene oxide terephthalate)-poly (butylene terephthalate) (PEOT/PBT) rods in goats and obtained fibrotic tissue tubes after one month [180]. After decellularization, the DECM grafts possessed sufficient mechanical strength with a bursting pressure of 2382 ± 129 mmHg and a suture retention strength of 1.97 ± 0.49 N. In addition, the endothelialization and abundant expression of collagen were observed at 8 weeks after grafting.

#### 4.1.2. Self-Assembly

In this approach, vascular cells are placed in a 3D environment and organize themselves to form the biomimetic vascular structure with/without biochemical and/or mechanical stimuli. Three techniques have been used to facilitate vascular self-assembly up till now, including cell-sheet assembly, microtissue aggregation, and cell printing [181]. For the cell-sheet assembly strategy, engineered cell sheets were rolled over a mandrel to form tubular structure, in which the cell junction and produced ECMs were maintained to promote cell communications [182,183]. The deposition of ECM proteins, such as collagen, laminin, fibronectin, and elastin, was facilitated via in vitro culturing, as well as the remolding of grafts into native-like constructs with equivalent mechanical properties [183]. L’Heureux et al. engineered SMCs and dermal fibroblast monolayer sheets and rolled them around a mandrel for the successful generation of a bilayered vascular graft via self-assembly [184]. After dynamic conditioning for 8 weeks in vitro and seeding ECs within the lumen to form an endothelium, the tubular grafts showed a bursting pressure of 2600 mmHg, while the grafts exhibited bleeding and failure in vivo at 7 days postimplantation [184]. To optimize this technique, L’Heureux et al. used human fibroblasts and increased the in vitro dynamic conditioning to 28 weeks [47]. The scaffold-free engineered grafts were named Cytografts (Cytograft Tissue Engineering, Inc., Novato, CA, USA), and were used in a clinical trial with ten patients. The grafts showed promising results in the clinical trial; in spite of one patient having died for a cause not related to the graft and three failures due to dilatation, thrombosis, or aneurysms, the grafts had a 78% patency at 1 month and 60% patency at 6 months [185]. Cell sheets combined with synthetic polymer sheets were used to enhance the mechanical properties and handling of the grafts [186,187], and the micropatterning of the polymer sheets was carried out to obtain cell sheets with a predesigned cell alignment [188,189,190].

**Figure 9:**
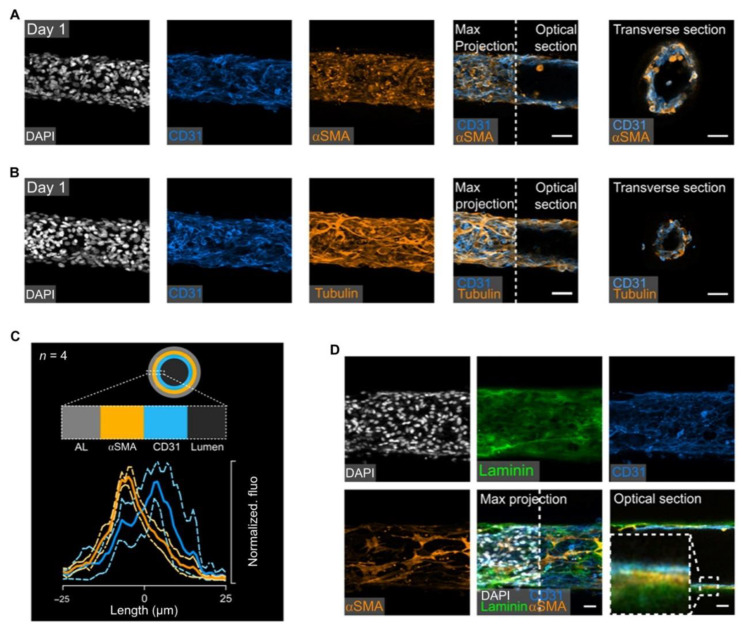
Self-organization of ECs and SMCs into artificial mature blood vessels. (**A**,**B**) Confocal imaging of engineered vessels immunostainings. (**C**) Fluorescence intensity distribution of αSMA and CD31. (**D**) Immunostaining images of laminin, CD31, αSMA, and DAPI of self-assembled vascular grafts. Ref. [195] Copyright 2019.

For the microtissue aggregation strategy, cells were encapsulated into an ECM mimicking a 3D environment with high density. Vascular-like constructs were formed via self-organization and the secretion of the ECM [191]. Kelm et al. fabricated a tubular graft through the aggregation of human-artery-derived fibroblasts and human umbilical vein endothelial cells (HUVECs); the accumulation of vessel-like tissue occurred within 14 days with an enhanced ECM expression and maturation [192]. The bioprinting strategy also embeds cells into an ECM mimicking ink, but deposits via the 3D-printing technique. Mironov et al. engineered a tubular graft through the fusion of printed cell spheroids [193], and Norette et al. developed a complex vascular tree with connected branches of accurate diameter and wall thickness [194]. Andrique et al. produced mature functional blood vessels by engineering hollow alginate hydrogel tubes internally coated with ECMs (Figure 9) [195]. The biomimetic configuration of lumens was achieved through the self-assembly of ECs and SMCs and perfusability, with contractility in response to vasoconstrictor agents being observed after the vesseloids reached homeostasis. Nevertheless, most bioprinted self-assembled vascular grafts are proof-of-concept for now, with the lack of mechanical properties and long-term stability hindering them from clinical applications.

### 4.2. Engineering-Based Techniques

#### 4.2.1. Electrospinning

**Figure 10:**
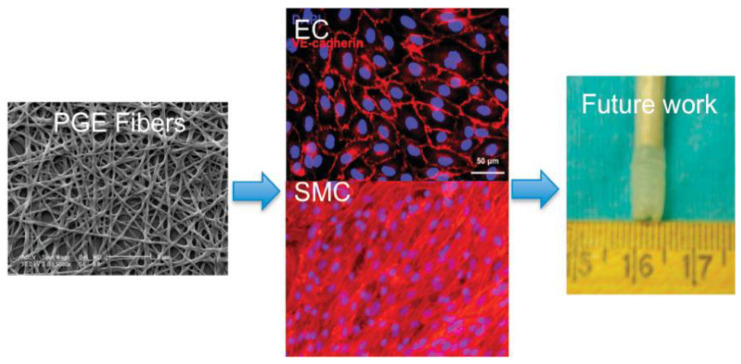
SEM images of hybrid PLGA–gelatin–elastin fibers (PGE fibers), immunofluorescence images of EC and SMC cultured on PGE fibers, and image of PGE fiber vascular grafts. Ref. [208] Copyright 2011, American Chemical Society.

In electrospinning, polymer solutions are dispersed through a strong electrical field onto a grounded or oppositely charged metal collector, creating continuous nano-to-microscale fibers for the formation of a thin film [196,197]. The diameter, morphology, alignment, and thickness of the electrospun fibers can be adjusted through the parameters, including the flow rate, solvent system, surface tension, supplied voltage, tip-to-collector distance, humidity, and temperature [198,199,200,201,202]. For the fabrication of engineered vascular graft, fibrous scaffolds are collected either on a rotating mandrel or, subsequently, rolled into a tubular structure to mimic the structure of native blood vessels [203]. Synthetic polymers, such as PCL, PLA, PGA, PLCL, poly (lactic-co-glycolic acid) (PLGA), poly (carbonate urethane) (PCU), and polyurethane (PU), and natural polymers, such as collagen, gelatin, chitosan, elastin, silk fibroin, and lecithin, have been used for vascular scaffold fabrication through electrospinning [35,144,204,205,206,207,208]. The electrospun grafts of natural polymers possess better biocompatibility, but inferior mechanical properties, while scaffolds fabricated with synthetic polymers exhibit comparable mechanical strength to native blood vessels, but a lack of bioactivity for cell adhesion, proliferation, and, development [35,204,209]. The electrospinning of blended natural and synthetic polymers has been used to fabricate vascular grafts with both superior biocompatibility and mechanical strength. By optimizing each component’s ratio in hybrid polymers, the presence of synthetic polymers, such as PCL, PLGA, or PLCL, helped retain the superior mechanical properties without interfering with the cell viability (Figure 10) [208,210].

Advanced electrospinning technologies, such as coelectrospinning, simultaneous electrospinning/electrospraying, sequential electrospinning, coaxial electrospinning, and sacrificial electrospinning, have been used to fabricate grafts with more complicated structures and superior properties [211]. Pan et al. tailored the degradation rates to better match in vivo cell infiltration and ECM deposition by adding a slow-degradation PCL into fast-degradation polydioxanone fibers via coelectrospinning [212]. Hyaluronic acid–gelatin nanoparticles were deposited simultaneously during PCL/collagen nanofiber electrospinning by Ekaputra et al., resulting in the enzymatical degradation of the nanoparticles creating pockets that improved cell infiltration [213]. Wang et al. developed an asymmetric vascular graft, of which the inner layer exhibited anticoagulant properties, and the outer layer showed antibacterial properties due to the sequential electrospinning of an inner layer of PCL/carboxymethyl chitosan and an outer layer of PCL/chitosan [214]. A gelatin shell was fabricated out of a synthetic polymer core using the coaxial electrospinning technique to enhance the proliferation of ECs and SMCs [113]. Moreover, bioactive polymers could be embedded in the core structure, performing a sustained release after implantation [215,216]. Sacrificial electrospinning has been used to create micro-to-mesovasculature networks. Water soluble fibers, such as polysaccharide pullulan and poly(N-isopropylacrylamide) (PNIPAM) fibers, were electrospun and embedded in bulk ECM-mimicking scaffolds, and after the dissolution of the fibers, perfusable channels with diameters from 1 to 55 μm could be obtained [217,218,219].

#### 4.2.2. Molding

Both tubular-shaped grafts and vasculature with interconnected branching structures can be developed through molding. In this approach, a polymer solution is cast into a hollowed-out mold and removed after solidification in the predesigned architecture [25,131]. A customized structure can be facilely produced by altering the geometry of the mold [220]. Tubular scaffolds can be fabricated with a simple device being set up, including an annular mold with an internal rod. The luminal diameter and thickness of the wall can be altered through the combination of differently sized annular molds and internal rods. However, the mechanical strength of the molded vascular grafts is relatively low compared to native blood vessels [221]. Strategies such as applying in vitro dynamic conditioning to cell-laden scaffolds, compacting the grafts through centrifugal forces, and the incorporation of porous meshes have all been used to strengthen molded tubular structures, especially those composed of natural polymers [222,223]. Associated with pore-generating techniques, the porosity of the scaffolds can be tuned to enhance cell infiltration. Lee et al. fabricated a PGS graft with a 75–80% porosity through salt leaching, which allowed for increased cell infiltration and ECM remodeling [224]. When combined with an inserted mesh or electrospun coating, this can reinforce the wall of the molded vascular grafts [225], while cell adhesion and orientation can be altered through surface patterning [226].

Sacrificial molding has been used to develop mesovasculature. The mold is firstly fabricated by using sacrificial polymers and then embedded within an ECM-like mesh [64]. After removing the mold through physical separation or dissolution, the ECM-like scaffold is left with interconnected mesochannels [64]. Chen et al. cast a 3D-reconstructed PLA filament in polydimethylsiloxane (PDMS) and, subsequently, dissolved it, producing vessel channels with endothelium through EC seeding [227]. In this work, blood vessel networks from different tissues, from mice to humans, have been successfully transcribed to physical microfluidic devices. Nevertheless, vasculature fabricated via this approach usually remain the bulk of ECM-like scaffolds, limiting their application in the theoretical study of the transportation of blood vessels, although not suitable for clinical applications.

#### 4.2.3. 3D Printing

Three-dimensional printing is another technology used in tissue engineering that has drawn increasing attention over the past decades. It is an additive manufacturing method in which inks are deposited layer-by-layer until the formation of predesigned computer-aided design (CAD) models. Extrusion, inkjet, and stereolithography are the three most used strategies for vascular graft development [6].

Extrusion-based printing is the most popular bioprinting technique that uses a fluid dispensed through mechanical or pneumatic actuation [228,229]. The technique can be applied to form large-scale 3D constructs with inks of varying viscosities, while the resolution is restricted in the range of 200–1000 μm, with tradeoffs present between printability and biocompatibility [230]. Vascular scaffolds can be fabricated with thermoplastics via fused deposition modeling; however, the high temperature required can deactivate bioactive molecules and damage cells [231]. As a result, acellular grafts have been fabricated with subsequent surface modifications to improve their hemocompatibility [232,233]. Instead, nature-derived polymers, such as alginate, gelatin, and fibrin, have been used for cell-laden vascular graft printing [234]. Tubular structures can be fabricated using layer-by-layer deposition through the repeated circular movement of the printer nozzle or direct extrusion in tubular architecture via coaxial needles. Two or more layered concentric tubes can be developed in a one-step process by applying coaxial printing. For example, alginate tubes can be fabricated via the coaxial printing of a CaCl_2_ core surrounded by a sodium alginate shell, while a GelMA tube can be obtained by extruding a gelatin core surrounded by a GelMA shell and removing gelatin by increasing the temperature after photocrosslinking [235,236,237].

Inkjet-based bioprinting is a noncontact printing technique, in which cell aggregates are firstly dispensed and then deposited onto a substrate, forming a vascular structure through self-assembly [238,239]. Even though inkjet-based bioprinting usually possesses a better resolution and faster printing speed compared with extrusion-based techniques, the required limited cell density and use of low-viscosity bioink, to avoid nozzle blockage, often result in a relatively weak mechanical strength [240]. Laser-assisted bioprinting and deposition into a reservoir with crosslinkers have been used to strengthen printed scaffolds, and complex structures (such as zig-zag tubes and vasculature with both horizontal and vertical bifurcations) and vascular scaffolds with increased cell density can be produced, respectively [241,242,243].

**Figure 11:**
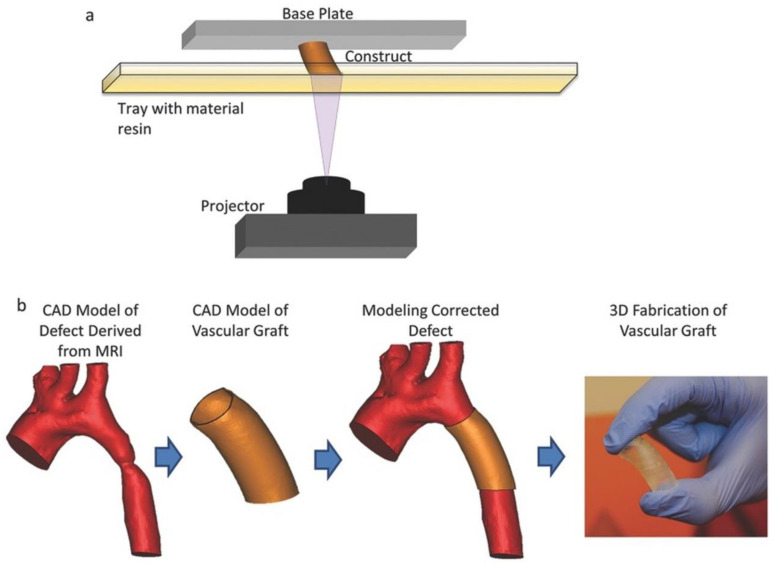
Fabrication of customized blood vessel grafts using 3D printing technology. (**a**) Digital light processing (DLP) stereolithography. (**b**) Design and fabrication process of 3D-printed graft to treat a coarctation of the aorta. Copyright 2015, Wiley-VCH.

Stereolithography-based bioprinting uses digital micromirror arrays to precisely control the light irradiation of the printed area in which photo-sensitive bioinks accumulate layer-by-layer through photocrosslinking [244]. Both acellular and cellular modes work for this approach. Stereolithography has its advantages in the throughput of vascular grafts with complex geometries in high resolution, while long exposure to UV light during the curing process may potentially damage cell viability [245,246]. Melchiorri et al. transcribed a recipient’s specific curvature into a customized vascular graft through stereolithography-based printing, which sustained the patency and functionality up to 6 months after implantation (Figure 11) [245].

#### 4.2.4. Laser Degradation

**Figure 12:**
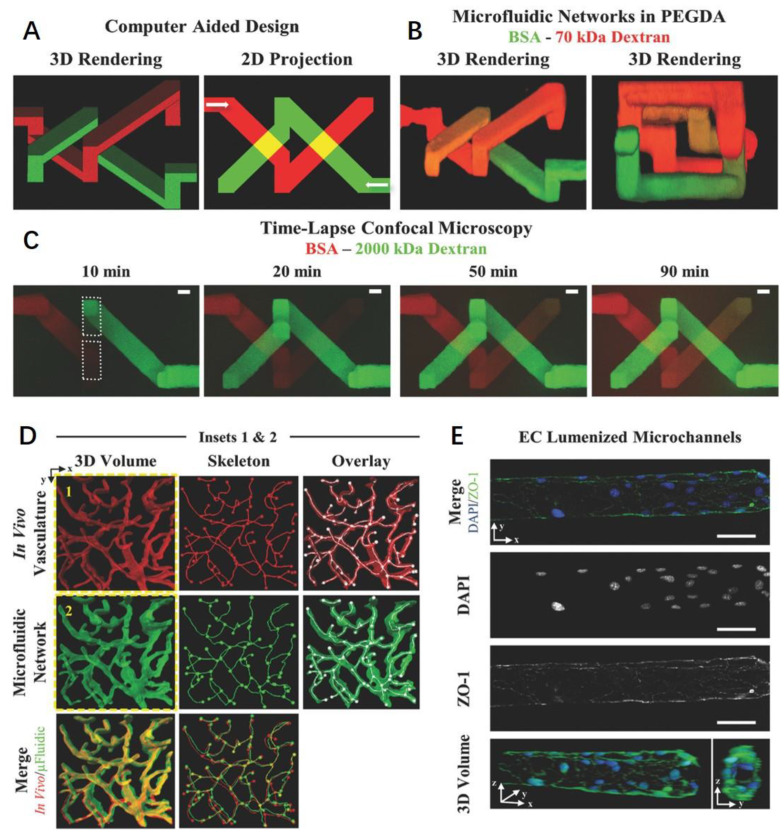
Three-dimensional biomimetic microfluidic networks in hydrogels fabricated with laser degradation. (**A**–**C**) Transport between 3D intertwining microchannels. (**D**) Dense, tortuous vasculature engineered in PEGDA. (**E**) Microvasculature endothelialized with ECs. Ref. [247] Copyright 2016, Wiley-VCH.

Laser energy has been used to selectively degrade or ablate predetermined regions within hydrogels with a two-photon absorption profile, including natural proteins, such as collagen, elastin, silk, high-protein-content hybrid materials (e.g., PEG–fibrinogen, PEG–albumin, and PEG–gelatin), PEG, and PEGDA [247,248,249,250,251,252]. High-resolution patterned microvasculature with mm scale z-depth penetration can be fabricated using laser degradation with/without cell encapsulation [6]. Brandenberg et al. demonstrated it by fabricating microchannels in a collagen hydrogel with a 50 μm diameter [251]. Heintz et al. combined a photodegradation hydrogel and image-guided laser control to generate a complex microvasculature that accurately recapitulated the in vivo vascular architecture and possessed a diameter size down to 3 μm (Figure 12) [247]. Intraluminal topography can also be produced through the use of the laser degradation method, and a complete endothelium can be acquired to promoted cell adhesion, migration, and proliferation [253].

## 5. Vascularized Tissue Engineering

The vasculature plays a fundamental role in the supply of blood, oxygen, and nutrients, and removing metabolic wastes for the maintenance of the function of all tissues, except the corneas, cartilage, and skin epithelium. A physiologically relevant tissue comprises of at least 1 × 10^8^ cells/cm^3^, which requires a high level of oxygen and nutrient supply [254]. The prevascularization of tissue-engineered grafts is preferred, especially for large-scale scaffolds. Peripheral nerves, bone, and the heart are tissues of which normal functions highly rely on blood supply. For example, a lack of blood supply in bone may lead to osteonecrosis, while the occlusion of blood vessels in the heart may lead to myocardial infarction. For this reason, vascularized tissue engineering scaffolds were designed for the regeneration of peripheral nerve, bone, and heart tissue. In this section, strategies for vascularized tissue engineering developments in peripheral nerve, bone, and heart tissue were summarized (Table 1).

### 5.1. Peripheral Nerve Tissue Engineering

The vascular system of peripheral nerves, also named vasa nervorum, can be categorized into two groups: the extrinsic system, which comprises a series of arteries and veins around the surface of peripheral nerves and supplies the epineurial and perineurial regions, and the intrinsic system, which is composed of small arteries and supplies the inner endoneurial nerve compartment [255,256]. While extrinsic vasculature can autoregulate in response to external physiological changes, intrinsic vessels lack this ability, and are susceptible to ischemia when the systemic blood pressure drops [257,258,259]. To minimize the interference induced by fluctuations in systemic blood pressure, peripheral nerve tissues are prone to low oxygen conditions with larger capillary distances in the vasa nervorum compared to other tissues [260]. Both extrinsic and intrinsic vasculature are important to nerve regeneration after injury. Blood vessels can proceed with Schwann cell migration and proliferation in the injured region, facilitate the formation of the Bungner bands, and guide axons growing from the proximal stump to the distal end. In addition, endothelial cells are able to secrete bioactive molecules, such as vitronectin, heparin sulphate proteoglycans, and the brain-derived neurotrophic factor (BDNF), to promote neurogenesis and nerve regeneration [261,262,263].

The first vascularized nerve graft was an ulnar nerve harvested by Strange et al. together with its blood supply [264]. Enhanced nerve regeneration in both length and size were obtained with vascularized nerve grafts, and Kanaya et al. demonstrated an improved sciatic function index (SFI) compared to the nonvascularized group in a rat sciatic nerve model [265,266]. However, autologous vascularized nerve grafts have limited resources and usually deteriorate the donor-site morbidity and scarring due to the removal of the local vascular supply, especially for large nerves. To overcome this disadvantage, nerve grafts were preimplanted between a host artery and vein for the generation of a microcirculation network. Ozcan et al. produced a vascularized amnion tube by embedding it between the femoral artery and vein for 3 weeks, subsequently using it to bridge a 1 cm long femoral nerve gap [267]. The vascularized amnion conduits exhibited superior performance in nerve regeneration compared to nonvascularized grafts in terms of the axon diameter, myelin thickness, and the number of myelinated axons. Iijima et al. demonstrated that PGA nerve conduits prevascularized with host superficial inferior epigastric vessels promoted remyelination and regeneration in a rat sciatic nerve injury model [268]. Nevertheless, a two-stage surgery was required in this approach, which prolonged the waiting time and could increase the risk of complications. Blood-vessel-including tabulation is another strategy for the fabrication of vascularized nerve grafts. In this approach, native blood vessels are wrapped with the nerve conduit. Kakinoki et al. bridged a sciatic nerve gap of 25 mm with a silicone tube encapsulating sural vessels in a rat model, demonstrating regeneration across the nerve gap and the reinnervation of the tibialis anterior muscle [269]. With the development of vascular tissue engineering, autologous blood vessels used in vascularized nerve grafts can be replaced with engineered small-diameter vascular grafts or micro- and mesoscale vasculature, avoiding the sacrifice of donor nerves and their blood supply, as well as the need for a two-stage surgery.

### 5.2. Bone Tissue Engineering

Blood vessels serve as a structural template in bone regeneration. Bone development takes place around them, and subsequent bone homeostasis is induced by them in the osteogenic environment [270]. The coupling of angiogenesis and osteogenesis occurs from skeletal development to postnatal bone repairing. The vascular endothelial growth factor (VEGF) is the master regulator for the effective coupling of angiogenesis and osteogenesis. During bone repair, the VEGF is produced by bone progenitor cells and osteoblasts to promote the migration and proliferation of ECs, while the ECs secrete osteogenic factors such as bone morphogenetic protein-2 (BMP-2) and insulin-like growth factor (IGF-1) to support osteoblast differentiation [271,272,273].

Several strategies have been used to develop synergistic vascularization and osteogenesis in engineered bone scaffolds, including ion doping, adding growth factors, altering topography, and the coculturing of cells [69,70,274,275,276]. Bioactive ions, such as Mg^2+^, Ca^2+^, Co^2+^, Cu^2+^, and Se^2+^, have been used as chemical inducers of the HIF-1α/VEGF signaling pathway to promote angiogenesis [277,278,279]. Various ion-doped materials have been fabricated, including bioglasses, metal–organic frameworks (MOF), metal scaffolds, and hybrid scaffolds, with synthetic polymers [280,281,282]. Improved vascularized bone formation has been demonstrated by Ma et al. on a Mg-doped tantalum scaffold in a rat femur condyle bone defect model [278]. The VEGF and its derived angiogenic peptides have also been introduced into bone grafts to enhance neovascularization. Li et al. loaded an angiogenic peptide QK into a GelMA-based injectable hydrogel, which exhibited synergistic osteogenic and angiogenic effects with a 58.2 ± 3.5% BV/TV ratio [283]. Furthermore, a mesoporous structure (2–50 nm) can not only promote the proliferation and differentiation of osteoblasts and ECs, but also increase the bone–matrix interface strength and stimulate mineralization [284]. The coculture of angiogenic cells with bone progenitor cells has been shown to have positive effects both on neovascularization and osteogenesis [285,286]. Zhou et al. reported enhanced capillary and bone formation in a BMSC/EC coculture system [287], and Wei et al. demonstrated cell–cell communication between human mesenchymal stem cells (hMSCs) and HUVECs taking place at a distance of ≤200 μm via paracrine secretion [288]. Despite current strategies for vascularized tissue engineering bone, prevascularized scaffolds for large defect repairs remain challenging, as the required microenvironment for osteogenesis and angiogenesis differs from mechanical strength to biochemical cues. Three-dimensional printing techniques can serve as potential solutions to this dilemma, although bioinks with adjustable mechanical strengths are required.

### 5.3. Heart Tissue Engineering

Cardiovascular diseases remain the leading cause of death, with heart failure potentially occurring due to the subsequent functional impairment of vasculature [289,290]. The coronary vasculature is crucial for cardiac development, homeostasis, and regeneration, as the heart is a highly vascularized organ where every cardiomyocyte is located close to a capillary [7,291]. ECs can regulate cardiac remodeling via angiocrine signals. For example, the cardioactive growth factor neuregulin-1 (NRG-1) released by ECs can promote cardiomyocyte proliferation and growth, as well as being capable of regulating the myocardial response to infarction and cardiomyocyte hypertrophy [292,293,294,295]; endothelin-1 secreted by ECs can regulate cardiomyocyte contractility and induce tissue remodeling [296].

**Figure 13:**
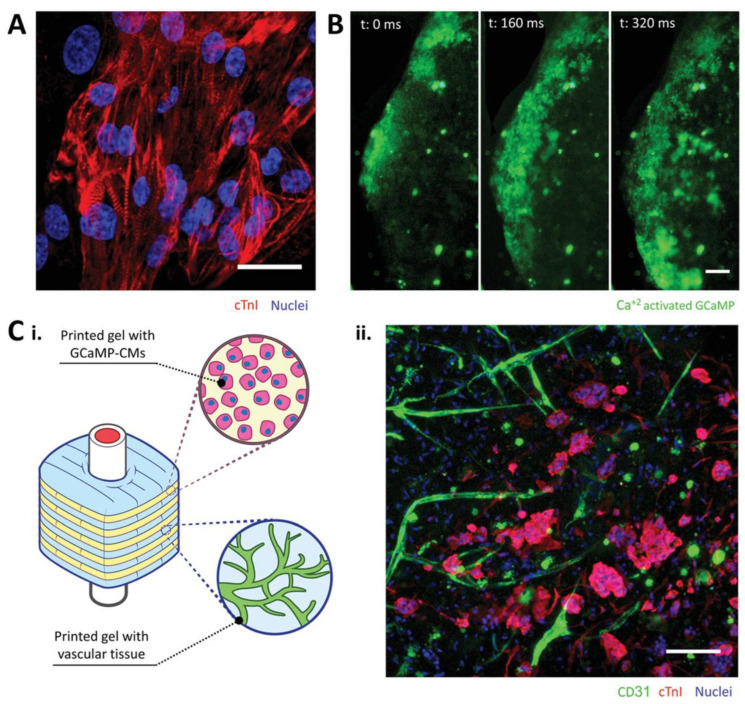
Cardiomyocytes supported with hierarchical vascular constructs. (**A**) Immunostaining of printed cardiomyocytes. (**B**) Spontaneous beat and electromechanical communication observed through calcium transients traveling distances over 1 mm. (**C**) Scheme of vascularized cardiomyocyte constructs and confocal image of vascular networks and cardiomyocytes. (**i**) schematic of vascularized cardiomyocytes constructs. (**ii**) immunofluorescence image of vascular network (CD31, green) and cardiomyocytes (cTnl, red). Scale bar = 100 μm. Ref. [20] Copyright 2021, Wiley-VCH.

The injection of stem cells and the implantation of cell-loaded or acellular scaffolds are the most used strategies for heart regeneration; however, the low cell survival rates and apoptosis due to a lacking oxygen and nutrient supply limit their clinical applications. Prevascularization is desired in cardiac tissue engineering, and connection between the engineered vasculature to the host is optimal for quickly supplying the implanted grafts. Coculture with vessel-forming cells is the most used prevascularization method at this time [297]. In this approach, endothelial cells and associated mural cells are cultured with cardiomyocytes and/or stem cells to promote vasculature formation [298,299]. Cocultured spheroids have been fabricated to provide suitable 3D structured for cell–cell and cell–matrix interactions, and interconnections to surrounding host blood vessels with blood perfusion were obtained [300]. Angiogenic growth factors, such as the VEGF, platelet-derived growth factor (PDGF), and fibroblast growth factor (FGF), have also been incorporated with synthetic, natural, or hybrid grafts to enhance the vascularization [301,302,303]. Moreover, microvascular tubes fabricated with cell sheet technology, as well as microvasculature developed via 3D printing or microfabrication, have been used for the prevascularization of engineered cardiac tissues. Well-perfused microchannels have been fabricated by coculturing ECs with cardiac cell sheets in a collagen hydrogel [304,305]. One strategy for prevascularization using 3D printing involves developing constructs with interconnected microchannel networks first, and then seeding with ECs, SMCs, and/or fibroblasts. Integration to the host’s vasculature can be achieved with this approach [306,307]. Another strategy is simultaneously bioprinting vasculature with surrounding cardiac tissue. Szklanny et al. engineered a hierarchical vasculature with millimetric tubular grafts and 3D-bioprinted vascularized tissue interconnections, and demonstrated their successful support of the in vitro functionality of cardiomyocytes (Figure 13) [20].

## 6. Conclusions and Perspectives

Engineered middle- and large-diameter vessels composed of nondegradable synthetic materials have been used for decades, while some biodegradable ones have reached the early stages of clinical translation. A comparable or better patency in long-term performance has been achieved, compared to autologous grafts. For small-diameter vascular grafts, acellular ECM-deposited products, such as Humacyte, have shown more potency in clinical applications and are currently under clinical trials. Nevertheless, most other works stay in the preclinical stage. Advanced biofabrication technologies have brought increasing vasculature of both micro- and mesoscale in recent years. However, limited results for the associated prevascularized engineered tissues have been reported. The lack of a comprehensive understanding of the specific vascular biology in each tissue, along with limited fabrication strategies for high-resolution large-scale scaffolds, are some of the road-blocks preventing the incorporation of established vasculature with engineered tissues. However, we believe that further development in materials science, stem cell biology, and biofabrication technologies can advance the engineering of functionally and hierarchically prevascularized tissues to off-the-shelf applications.

## Figures and Tables

**Table 1:** Vascularized tissue engineering.

Tissue	Strategy	General Functions	Examples of Studies	References
Peripheral nerve	Autologous grafts	Enhanced regeneration in nerve length, size, and sciatic function index (SFI)	Ulnar nerve with its blood supply	[264–266]
	Preimplantment of nerve grafts to obtain microcirculation network	Enhanced axon diameter, myelin thickness, and the number of myelinated axons	Vascularized amnion tube obtained through embedding between the femoral artery and vein	[267,268]
	Blood-vessel-including tabulation	Regeneration across the nerve gap	Native blood vessel wrapped with the nerve conduit	[269]
Bone	Ion doping	Promoted angiogenesis through HIF-1α/VEGF signaling pathway	Mg-doped tantalum scaffold	[277–282]
	Adding growth factors	Improved neovascularization and synergistic osteogenesis	GelMA-based hydrogel with angiogenic peptide QK	[283]
	Altering topography	Promoted proliferation and differentiation of osteoblasts and ECs; increased bone–matrix interface strength and stimulate mineralization	Pore-structured scaffolds based on poly(3-hydroxybutyrate-co-3-hydroxyhexanoate) (PHBHHx)	[284]
	Coculturing of cells	Enhanced capillary and bone formation	BMSCs/ECs coculture	[285–288]
Heart	Coculturing of cells	Promoted vasculature formation	Endothelial cells cultured with cardiomyocytes	[297–300]
	Adding growth factors	Enhanced vascularization	VEGF	[301–303]
	Microvascular tubes fabricated with cell sheet	Formation of well-perfused microchannels	Coculturing ECs with cardiac cell sheets in a collagen hydrogel	[304,305]
	Prevascularization through 3D printing	Integration to the host’s vasculature	A hierarchical vasculature supporting in vitro functionality of cardiomyocytes	[20,306,307]

## Data Availability

Data sharing is not applicable to this article as no new data were created or analyzed in this study.
